# Acute aortic dissection mimicking a gastric ulcer: Medico‐legal implications

**DOI:** 10.1002/ccr3.2315

**Published:** 2019-07-21

**Authors:** Med Amin Mesrati, Marwa Boussaid, Nouha Ben Abdejlil, Abdelfeteh Zakhama, Abir Aissaoui

**Affiliations:** ^1^ Department of Forensic Medicine Taher Sfar Hospital Mahdia Tunisia; ^2^ Department of Cytology and Pathology Fattouma Bourguiba Hospital Monastir Tunisia

**Keywords:** aortic aneurysm, autopsy, legal, liability, sudden death

## Abstract

The diagnosis of aortic dissection is often difficult because the symptoms are usually non‐specific. AD should be considered in the differential diagnosis of all cases of idiopathic retrosternal pain. Misdiagnosis of AD is a common cause of legal suits in medical practice. Prevention requires a complete and thorough evaluation.

## 1 INTRODUCTION

Aortic dissection is an emergency condition. It is defined as a disruption of the medial layer provoked by intramural bleeding, resulting in separation of the aortic wall layers and subsequent formation of a true lumen and a false lumen with or without communication.^1^ The Acute dissection is defined as occurring within 2 weeks of onset of pain.^2^ It is an uncommon life‐threatening cardiovascular emergency. The clinical manifestations are frequently non‐specific. It can mimic the onset of many other conditions including those involving the abdominal system.^3^ Thus, the diagnosis is often difficult, challenging and death may occur.^4^ In this situation, the medico‐legal implications are far‐reaching. In fact, the doctor can be liable for diagnostic errors.

Herein, we report an autopsy case of aortic dissection mimicking gastric ulcer and we discuss its medico‐legal implications.

## 2 CASE PRESENTATION

**Figure 1:**
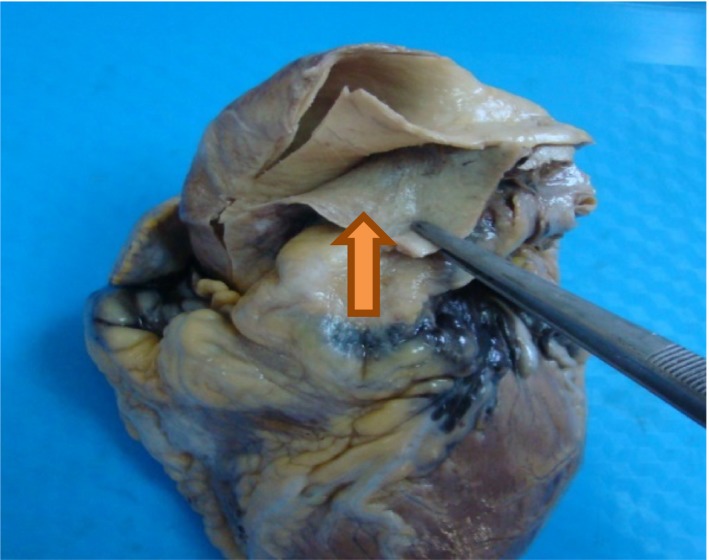
Macroscopic view of the heart (after formalin fixation) showing a dissection of the ascending aorta (the arrow shows the false lumen)

**Figure 2:**
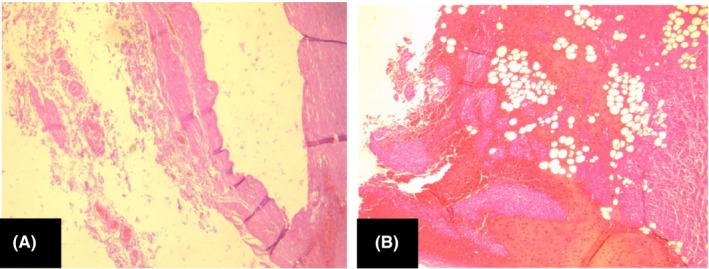
A, Aortic dissection with false lumen between the middle and outer thirds of the media HEx40; B, Extravasation of blood outside the adventitia HEx40

A 45‐year‐old female consulted the emergency room for headache associated with retrosternal pain radiating to the epigastrium, for which she had a symptomatic treatment. She had a Past medical history of Peptic Ulcer disease with recurrent epigastric pain. Additionally, she had no past history of hypertension, congenital cardiovascular malformations, or trauma. Regarding social history, the patient did not have any history of tobacco or alcohol use. She had no family history of sudden cardiac death. The next days she revisited the Emergency Room for the same symptomatology with an exacerbation of the epigastric pain. The electrocardiography was normal. Chest X‐ray did not show any abnormalities. The blood pressure was at 140/80 mm Hg. The laboratory tests (cardiac markers) showed no disturbances. The diagnosis of myocardial infarction was ruled out, and the diagnosis of hyperalgic Peptic Ulcer was retained. She had an injection of PPIs (Proton pump inhibitors) which relieved her, and she returned home with a symptomatic treatment based on PPIs. A few hours later, she was discovered dead on her bed. A forensic autopsy was ordered. No external injuries were found on the body. On internal examination, dissection of the thoracic stage revealed a large abundant hemopericardium. The lungs were edematous. The heart weighted 380 g. The examination of the aorta showed type II DeBakey aortic dissection (Figure 1). The other organs were congested. The histological examination confirmed the diagnosis and showed a dissection of the media (Figure 2). The toxicological screening was negative.

## 3 DISCUSSION

Acute aortic dissection (AAD) is a rare and serious cardiovascular disease more commonly reported in elderly males. The incidence of AAD is estimated at six per 1000 persons per year.^5^ It is higher in men than in women and increases with age.^6^ In a large contemporary Swedish population study, the incidence of thoracic aortic disease was 16.3 per 100 000 per year in men and 9.1 per 100 000 per year in women.^6^Research has found multiple risk factors that may increase AAD occurrence such as atherosclerosis, hypertension, connective tissue disorders, Marfan's syndrome, congenital cardiovascular malformations, cystic medial necrosis, and pregnancy.^7^ However, it has also been reported in young woman without any risk factors as in the above case.^8^ The prognosis is poorer in women, as a result of atypical presentation and delayed diagnosis.^1^This condition requires immediate diagnosis and adequate treatment. It is a deadly disease. In fact, Erbel et al^9^ showed that the mortality rate of AAD increases of about 1%‐2% per hour if the patient is not treated during the first 48 hours after the onset of the clinical manifestations. It is frequently misdiagnosed on initial assessment because a timely diagnosis is often difficult and challenging. The incidence rate of patients with misdiagnosed AAD is reported to be 16%‐38%.^10^ Spittel et al^11^ reported that 28% of patients with aortic dissection had been misdiagnosed until postmortem examination.

In the literature, there have been multiple studies dealing with this subject. They studied the risk factors that lead to failure to diagnose AAD in the emergency room.^4^, ^10^ Kurabayashi et al^10^ reported three factors leading to misdiagnosis, namely a mild clinical presentation that is not evocative of a severe disease, mimicry of another condition, and absence of typical clinical or paraclinical findings. In fact, AAD symptoms are variable. The main symptom, reported in literature, is still a retrosternal pain in proximal dissections and interscapular or back pain in the distal dissections.^9^ However, the frequency of this typical symptom is low. Some related studies have found that the frequency of painless AAD is about 5%‐15%.^12^, ^13^ In fact, it can mimic other conditions such as acute myocardial infarction, coronary artery disease, cholecystitis, acute gastroenteritis, stroke, and hyperalgic ulcer. Thus, the diagnosis can be misinterpreted, as in the present case. In fact, the patient presented with retrosternal pain radiating to the epigastrium. A history of peptic ulcer disease with multiple repeated hyperalgic episodes raised a suspicion of ulcer pain, and she was sent home with a symptomatic treatment. The patient however, did not have any history of cardiovascular illnesses. Chest radiography and electrocardiography can give the first clues to diagnose AAD, but they have a low sensitivity and specificity.^14^ Multiple studies have suggested D‐Dimer should be used as a positive biomarker in diagnosing AAD.^15^ However, it may not always show in patients with acute aortic dissection if there is a lack of communication between true and false lumen. In recent years, several modalities have been developed and have been widely used to diagnose AAD with high accuracy, particularly by radiological methods, including computed tomography (CT), transesophageal echocardiography (TEE), and magnetic resonance imaging (MRI).^16^ However, they are not usually ordered since the diagnosis is not even suspected, as in the reported case.

In cases of misdiagnosis, the medico‐legal implications could be far‐reaching and the doctor may be held responsible. Most current claims involve doctors at the front lines who examined a variety of situations where the diagnosis may be misinterpreted.^17^

The main factors that lead to AAD misdiagnosis in the Emergency Department are failure to perform adequate history taking or/and physical examination, failure to identify atypical symptoms, failure to order or to interpret a diagnostic test and failure to order an appropriate specialized consultation.^18^ Malpractice law suits about AAD misdiagnosis can be subject to trials in both penal and civil judgment. In fact, the civil medical liability of the doctor can be engaged and they will be required to pay compensation to the victim or their relatives if death occurs. This situation requires the association of damage, a fault and a causal link between the fault and the damage. The medical civil responsibility can be covered by professional insurance for doctors or by medical establishments.

Furthermore, the doctor would also be held responsible in criminal law. The physician can be prosecuted under section 225 of the Tunisian Penal Code (TPD) for causing involuntary assault by negligence or inattention, with a prison sentence that may extend to 1 year. If death occurs, the doctor can be prosecuted for involuntary homicide under section 217 of the TPD and can be sentenced to prison for a period that may extend to 2 years.

Failure to diagnose AAD can be prevented by maintaining standard recommendations. The key to this disease management is to maintain a high level of vigilance and suspicion for this diagnosis.^19^ Doctors should be more aware and identify atypical presentations. John et al^17^ recommended that physicians on the front lines should be encouraged to suspect aortic diseases in all cases involving chest pain or unexplained abdominal pain or hypotension and to use D‐Dimer as a screening tool for aortic dissection. Furthermore, CT or MRI scan should be undertaken as soon as possible, which would exclude the three major killers: myocardial infarction, pulmonary embolism, and acute aortic dissection. Wang et al^20^ proposed that all the patients with acute chest pain should receive an ECG and echocardiography at the time of first medical contact. The American College of Cardiology (ACC) and American Heart Association (AHA) guidelines for the diagnosis and management of patients with thoracic aortic disease proposed the aortic dissection detection risk score system, to detect AAD early and rapidly in patients presenting with acute chest pain, based on predisposing conditions, pain features, and clinical examination.^21^ Recently, researchers investigated values of combined use of the risk score and the ascending aorta diameter >40 mm for the early identification of AAD type A.^22^ They found that combined use of an aortic dissection detection risk score ≥1 and an ascending aorta diameter >40 mm was highly indicative of AAD type A in patients presenting with acute chest pain, especially in patients with Acute myocardial infraction secondary to AAD. This situation requires performing urgently computed tomography angiography (CTA) or magnetic resonance imaging (MRI) to confirm the diagnosis of A‐AAD.

## CONFLICT OF INTEREST

None declared.

## AUTHORS' CONTRIBUTION

MAM: conceived the presented idea. MB: wrote the manuscript. NA: illustrated the figures. AZ: contributed to the final version of the manuscript. AA: contributed to final version of the manuscript.
